# A framework for generative AI-driven extraction of clinical user needs in pediatric device development

**DOI:** 10.3389/fdgth.2026.1726098

**Published:** 2026-04-14

**Authors:** Abdelrahman Abdou, Niraj Mistry, Douglas M. Campbell, Sridhar Krishnan

**Affiliations:** 1 Department of Electrical, Computer and Biomedical Engineering, Toronto Metropolitan University, Toronto, ON, Canada; 2 Department of Pediatrics, The Hospital for Sick Children, Toronto, ON, Canada; 3 Department of Pediatrics, St. Michael’s Hospital, Unity Health, Toronto, ON, Canada

**Keywords:** GenAI, LLM, neonatal resuscitation, pediatric, user needs

## Abstract

**Introduction:**

Generative artificial intelligence (GenAI) is becoming an important tool in medical product development. A main component of this development includes annotating, summarizing, and extracting key insights from expert interviews to identify clinical pain points and curate device requirements. These tasks are time- and labor-intensive, resulting in increased administrative burden and reduced efficiency. As a result, researchers have developed large language models (LLMs) that can disseminate research and interview findings with reduced workload and improved productivity. This study explores the use of GenAI, specifically GPT-4o, to extract user functional and design requirements from medical professional interviews for the iterative development of an infant heart rate detector for neonatal resuscitation.

**Methods:**

A total of 29 healthcare practitioners were interviewed using a semistructured interview format. The interviews were recorded and transcribed. GPT-4o was used to extract user insights from the transcripts, and the results were compared with manual interviewer notes.

**Results:**

A total of 26 h of interview data were collected. All interviewees validated the clinical need for a modality that enables quick and accurate heart rate (HR) measurement during neonatal resuscitation. A set of user requirements was extracted from the interviews and curated under the themes of ease of use, fast and accurate HR measurement, reusability, display, battery life, start-up time, and cost. Also, quantitative analyses of the interviewee’s years of experience, clinical settings, and specialties were conducted.

**Discussion:**

These analyses were conducted using GPT-4o and compared with ground-truth manual annotations to determine the accuracy and reliability of GenAI in content extraction and summarization.

**Conclusion:**

Overall, this study explored the user requirements identified through in-depth interviews for the development of a pediatric medical device. It also aimed to demonstrate the potential of GenAI in curating these design requirements, offering a framework for researchers and product designers to explore the use of LLMs in curating user requirements and design specifications for medical devices.

## 1 Introduction

Over 10% of newborns worldwide require breathing assistance within the first minute after birth each year (1). Approximately 1% of newborns require more extensive resuscitation interventions. Currently, pediatricians rely on auscultation using a stethoscope, electrocardiography (ECG), and pulse oximetry (PO) to determine heart rate (HR), which guides neonatal care protocols for resuscitation and breathing support interventions (2). Unfortunately, these modalities are inaccurate or too slow to provide HR information within the first minute after birth, also known as the golden minute. This critical interval requires interventions to occur within the first minute after birth to avoid neonatal hypoxic injury and improve the newborn’s chances of survival during respiratory distress.

Auscultation is considered the fastest approach to acquire HR. Clinicians estimate HR by counting the number of heartbeats over 6 s and multiplying by 10 to obtain beats per minute. However, this approach produces unreliable HR estimates because it is operator-dependent and prone to human error. Such inaccuracies can affect the clinical decision-making process for neonatal resuscitation program (NRP) interventions (2, 3). Pulse oximetry (PO) offers higher accuracy than auscultation but is limited in time-sensitive scenarios such as neonatal resuscitation. PO can take up to 1 min or longer to acquire a reliable HR reading (4–6). PO relies on photoplethysmography (PPG), which measures blood flow to compute HR. Due to low peripheral perfusion in neonates immediately after birth, PO may require extended time to provide accurate HR measurements, thereby failing to meet the requirements of the golden minute. Furthermore, PO can be an unreliable estimator of HR in the presence of motion artifacts, variable skin pigmentation, and poor peripheral perfusion (7). ECG, considered the gold-standard approach for HR detection in neonatal resuscitation, may take a few minutes to provide reliable HR measurements (6, 8, 9). ECG requires correct placement of wet adhesive ECG electrodes on the infant, which is time-consuming and can delay accurate HR estimation during neonatal resuscitation. These limitations across all above-mentioned modalities have driven the development of a novel infant heart rate detector (HRD) that is fast, accurate, and capable of displaying HR within 10 s of contact with the infant using single-lead ECG 3D-printed dry electrodes (10–12).

On the other hand, large language models (LLMs) are GenAI-based conversational tools that offer significant benefits to users. GenAI refers to the use of AI to generate new content based on training datasets (13). LLMs are designed to understand and generate this content in relation to human language. These GenAI models are trained on large amounts of text and information, including data from the internet, books, articles, conversations, and other text-based sources (13). LLMs are designed to identify patterns within these large datasets using an autoregressive probabilistic language modeling approach (14, 15). The most prominent LLM to gain rapid popularity since 2023 is ChatGPT (OpenAI) (16). ChatGPT is an accessible LLM build on pretrained transformers and further fine-tuned for conversational use (14). Users can prompt ChatGPT to ask questions and converse with it. The prompts and responses are later used in successive replies by ChatGPT to provide better context and information for user queries. LLMs, through their conversational interface, have enabled users to integrate GenAI into their everyday activities, including asking in-depth questions, performing administrative tasks, and seeking advice. This tool not only features conversational attributes but also performs many tasks, including code generation, text summarization, translation, documentation generation, image creation, and personal assistant tasks. For example, Sasseville et al. performed a systematic review to evaluate the advantages of AI scribes in reducing documentation burden and clinician burnout. Their work demonstrated that AI scribes have the potential to improve documentation efficiency and positively impact clinician workflow (17). Tierney et al. developed an AI scribe that transcribes patient–doctor encounters for over 10,000 clinicians and staff at The Permanente Medical Group (TPMG). Their findings showed that physicians using the AI scribe experienced reduced documentation burden and spent less time on electronic medical record (EMR) systems (18). Other notable LLMs that have shown potential and are extensively used by users globally include Gemini (Google), DeepSeek V3 (DeepSeek), Claude (Anthropic), Grok (xAI), Llama (Meta), and GPT-4o (OpenAI). Each LLM utilizes its proprietary dataset, which contributes to variations in outcomes across tasks such as medical examinations, clinical documentation, and other clinical work (19–22).

Given the capability of LLMs to perform administrative tasks, many researchers have explored their use as support systems in clinical practice. Liu et al. demonstrated that ChatGPT can function as a clinical decision support system across many clinical applications, including cancer screening (23), clinical decision-making (24), differential diagnosis (25), and responses to complex medical queries such as those related to hepatic conditions (26) and common retinal diseases (27). Also, ChatGPT has been used to clinically summarize patient data, thereby reducing the burden associated with patient chart reviews (28). Lee et al. showed that LLMs have the potential to help clinicians reduce administrative tasks and burden, allowing more time for patient-centered interactions (29).

Other researchers have highlighted the potential of LLMs to qualitatively extract insights from interview transcripts (30–32). Previously, qualitative medical research required researchers to transcribe audio interviews to text (33). This process was followed by reading the transcripts and extracting important information from the interviews subjectively. Depending on the researcher and their experience, different insights could be derived from the same interview (32). In turn, this issue led to high variability due to personal bias and inconsistent reporting. Human bias in extracting interview insights led to limitations in determining patterns in responses across many interviews (33). These challenges have contributed to the development of LLMs for transcription and interview insight acquisition. With advancements in LLMs, transcription and insight extraction have become automated, minimizing workload and lessening administrative burden for researchers (28, 29, 31, 32, 34). These LLMs also facilitate easier pattern determination and recognition across many interviews. For example, Goyanes et al. used ChatGPT to extract key insights and perform thematic analysis in qualitative research (35). Their outcomes showed that their framework, which relied on ChatGPT, can be used to summarize and extract information from interviews (35).

**Figure 1:**
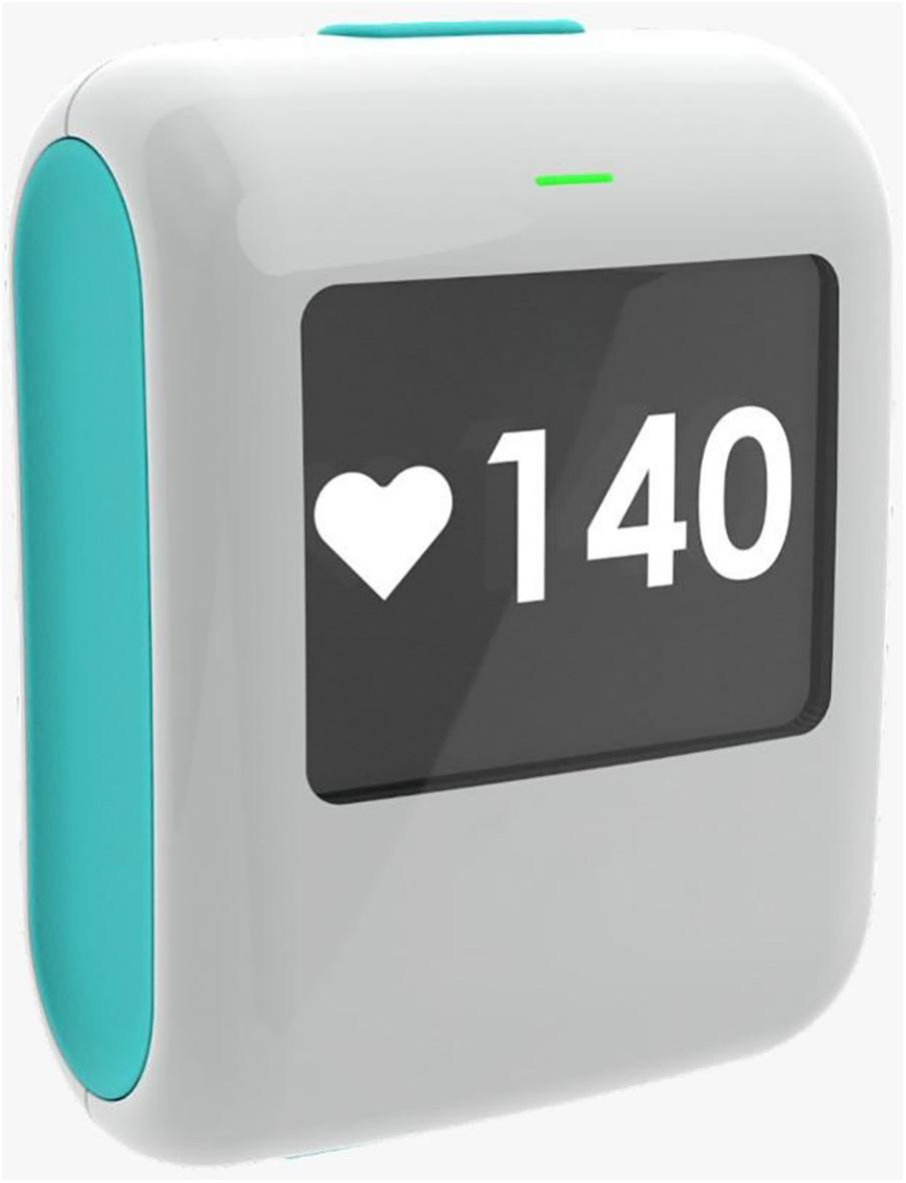
Infant HRD for quick and accurate HR measurements. The proposed infant HRD based on user requirements quickly displays HR within 10 s of initial neonatal contact.

This work is the first of its kind to explore the use of LLMs in curating user requirements for a pediatric medical device. This study explored the use of AI-based transcription and insight extraction to obtain user requirements for the iterative development of an HRD for neonatal resuscitation. Currently, neonatal resuscitation lacks an HR measurement tool that is both accurate and rapid to provide HR measurements within the first minute of life. This study serves as a precursor to the device development process, in which user requirements were generated from clinician interviews, which led to the development of the final prototype HRD, as shown in Figure 1.

This work presents a structured framework that integrates clinician insights with LLM tools for early-stage requirements refinement and the iterative development of a medical device for neonatal care. Insights derived from clinical interviews were first used to validate the clinical need and ensure alignment with the stated clinical limitations in HR detection described in the literature. These insights were also used to design and validate the HRD device described above. It is important to note that the HRD was not initially designed solely based on interview data. However, initial HRD design concepts and early prototype iterations had already been developed to establish baseline functionality. Clinician interviews were then used to refine and prioritize user requirements. This approach ensured that the final prototype design better reflects real-world clinical workflows and usability expectations. The primary goal of this study was to extract clinician perspectives and translate these insights into the ongoing development and validation of an HRD for neonatal resuscitation. By iteratively developing a medical device technology with the end-user in mind, the design process focused on the device’s usability and clinical fit, which are key determinants of its long-term use and clinical acceptance. We used a user-centered design approach that ensured that clinical needs, workflows, and medical resource constraints were considered to increase the likelihood of widespread clinical adoption.

The proposed final infant HRD prototype was developed based on user requirements in this study. The device was investigated in 50 healthy newborns to determine its accuracy and reliability in HR detection (10, 11). The device showed good performance, revealing that the HRD can accurately display HR within 10 s of initial newborn contact (12).

This paper is structured as follows: the Methods section discusses interview preparation, transcriptional steps, and the prompts used with GPT-4o to summarize and extract qualitative and quantitative insights from interviewees. The Results and Discussion section then outlines the user requirement insights obtained from the interviews. Finally, the Conclusions section provides the concluding remarks for this study.

## 2 Methods

### 2.1 Study design

We performed a qualitative study to identify the user requirements for the iterative design and development of an infant HRD for neonatal resuscitation. Our proposed infant HRD was developed in alignment with these needs. In this work, we followed the Consolidated criteria for Reporting Qualitative research (COREQ) checklist in both conducting the study and curating the outcomes, as outlined in the Supplementary Material (36). We interviewed 29 medical professionals, including 12 neonatologists, 10 general pediatricians, three pediatric nurses, two obstetricians, one midwife, and one NICU manager. We chose to conduct detailed semistructured interviews over focus groups and surveys to obtain a diverse range of feedback and individual perspectives from each participant.

A questionnaire was designed to guide the open-ended semistructured interview conversations. This approach allowed us to collect both quantitative and qualitative data regarding the design and functionality requirements of the infant HRD. A high-level HRD concept was included in the questionnaire to guide interviewees and facilitate open-ended exploration of clinicians’ perspectives, enabling them to agree or disagree with proposed features, identify missing elements, and suggest additional requirements. The questionnaire is provided in the Supplementary Material. Each interview was designed to last between 45 min and 1 h.

### 2.2 Study setting

Interviews were conducted through an online conference platform (Zoom) and were recorded. The locally stored recordings were transcribed using Tactiq.io (Sydney, Australia), a GDPR-compliant transcription software that does not store visual or audio data after transcription. A principal interviewer, a pediatrician–researcher, conducted all interviews. The researcher was experienced in conducting interviews with pediatric stakeholders due to his role as a physician–scientist, interacting with patients, other clinicians, hospital administrators, medical students, and medical technology company representatives. This researcher identified the clinical need in question: the lack of effective and accurate HR measurements within the first minute after birth. The researcher aimed to validate this clinical need and identify the user requirements for a new HR measurement modality that would support maximal clinical adoption. After the interview recording, interview transcripts were generated and stored in PDF format. The transcripts were later anonymized by a graduate student by removing all information that could identify specific speakers. The transcripts were reviewed and corrected by the principal interviewer.

### 2.3 Participant recruitment

Participants were recruited via email and through introductory referrals from previous participants. This approach enabled us to recruit medical professionals quickly and helped minimize the interview rejection rate.

### 2.4 Data collection

An automated generative AI prompt template was developed to extract the most relevant information about user requirements for the design and iterative development of an HRD from each interview. The predefined prompt is outlined as follows. Analyze the attached interview transcription and perform the following tasks:

1. Provide a summary of the interview.
2. Identify the speakers and their occupations.
3. Determine their total years of experience in their various roles.
4. How does the interviewee speaker feel about the stated problem?
5. What are their thoughts about an infant heart rate detector for the stated problem? (include their perceived advantages and concerns).
6. How can the infant heart rate detector be used? Provide a list of primary and secondary use cases.
7. Provide other important highlights that were not mentioned earlier.
8. Provide a final takeaway message.

These template prompts were designed to constrain the GenAI to consistently extract the most relevant information from each interview. Each task was prompted sequentially using a prompt chaining approach, which improves traceability by structuring the extraction into discrete steps. However, this process may introduce dependency effects, whereby earlier LLM responses influence later steps. We mitigated this risk by separating summarization tasks from downstream extraction. In other words, downstream questions, such as attitudes and use cases in tasks 5 and 6, were designed to prompt the LLM to directly extract this information from the transcript, which reduced the chance that later responses would repeat the phrasing from initial summaries. The approach encouraged the model to re-scan the transcript for different types of evidences rather than generalizing from earlier responses. Furthermore, the template included two open-ended tasks (tasks 7 and 8) to capture information that may not have been elicited by the previous predefined tasks. These specific tasks provided an explicit opportunity to retrieve additional details that may have been missed in earlier steps, thereby reducing the risk of information loss. The predefined prompt templates were implemented using GPT-4o (OpenAI, California). Each interview transcript was first processed using the abovementioned prompting technique. The second phase of GenAI prompting involved collecting all similarities and differences across all recorded interviews within this specified chat. GPT-4o is known to store chat information and use it in its future responses to prompts that instigate GPT-4o’s memory and retrieval mechanisms. This concept was being taken into consideration to investigate the GenAI’s memory allocation and information retrieval capabilities. It is important to note that this phase was susceptible to context carryover, which may affect the accuracy of summarized information. To test these mechanisms, the following prompt was used:

Based on the entire chat,

1. How many people were interviewed?
2. Specify each interviewee’s specialty and group them.
3. Provide a range of years of experience for all the interviewees.
4. Provide the interviewees’ primary clinical settings.

To determine the accuracy of information retrieval, the abovementioned tasks were also performed manually and recorded. GenAI chat information retrieval was compared directly with manual annotation ground truths. This methodology allowed us to determine the reliability and robustness of GPT-4o’s information retrieval capabilities.

### 2.5 Feature interest scores

Quantitative interest scores were obtained using a five-point Likert scale, where 1 = “not interested” and 5 = “definitely interested,” for each statement in the questionnaire (Supplementary Material). These scores were used to quantify each interviewee’s perceived importance of, and interest in, specific device features. While most features were predefined in the Supplementary Material, the semistructured interview format also allowed interviewees to propose additional features not explicitly mentioned. Across all interviews, the features mentioned and evaluated in this study included fast and accurate HR detection, hands-free use, audio output, visual display, reusability, portability/battery life, EMR integration, ease of use, cost, and start-up time, as summarized in Table 1. Average interest scores were then calculated for each feature across all interviewees.

**Table 1:** Device features.

Device features	Definitions
Fast and accurate HR detection	Refers to the prototype’s ability to provide a HR measurement within 10 s
Hands-free use	Refers to enabling operation without requiring both hands during neonatal resuscitation
Audio output	Refers to the presence of a sound to guide resuscitation steps
Visual display	Refers to the presence of a screen for viewing HR and other vital sign metrics
Reusability	Captures the ability to safely reuse the device across multiple neonates, including cleaning and infection-control considerations
Portability/battery life	Reflects the feasibility of using the device across different clinical settings and the expected duration of operation before recharging or replacement
EMR integration	Refers to the capability to interface with EMR for documentation and data capture
Ease of use	Reflects how quickly and reliably clinicians can learn and operate the prototype in practice
Cost	Refers to the anticipated purchase and/or implementation cost of the technology
Start-up time	Refers to the time required to power on and have the device ready for use

### 2.6 Ethical considerations

The user-requirements interviews reported in this manuscript were conducted with healthcare professionals as part of an early needs-assessment process to inform the design and development of the heart rate detector. The interviews informed device development study approved by the Scarborough Health Network Research Ethics Board (REB file no. PED-21-025). Participants provided written indication of consent by replying to the recruitment email, and verbal consent was reconfirmed at the time of the virtual interview. Interview transcripts were de-identified prior to analysis through removal of all direct personal identifiers such as name, age, gender and geographical location. The first author, who was not involved in the interview process, received only de-identified transcripts for qualitative analysis. Only non-identifiable qualitative data are reported in this manuscript.

## 3 Results

**Figure 2:**
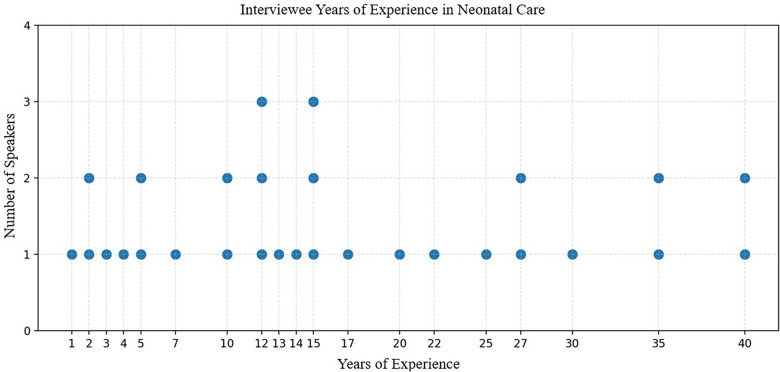
Dot plot of interviewee experience. The dot plot shows the range of experience, in years, among the 29 medical professionals in neonatal care.

A total of 26.45 h or 1,587 min of interview data were recorded, with each interview lasting an average of 55 min. All medical professionals had experience in neonatal resuscitation and infant delivery. Their levels of experience ranged from recently graduated fellows to clinicians with over 40 years of practice in neonatal care. Most practitioners had between 12 and 17 years of neonatal care experience, as shown in Figure 2.

Interviewees were categorized based on their primary clinical setting. A total of 23 medical practitioners work within urban hospital environments, while 4 and 2 physicians work in rural and community hospitals, respectively. All 29 professionals (100%) agreed that there was a lack of a reliable approach to quickly estimate accurate HR and other vital signs during neonatal resuscitation.

### 3.1 Interest scores

All 29 practitioners (100%) expressed support for the development of a single-lead ECG-based HRD for newborn HR assessment during neonatal resuscitation. A mean interest score of 4.5 out of 5 was highlighted for the development. Most participants reported interest levels between “very interested” to “definitely interested” in incorporating the HRD into their practice. Neonatologists alone showed the greatest eagerness for the HRD, with a mean interest score of 4.7 out of 5.

**Figure 3:**
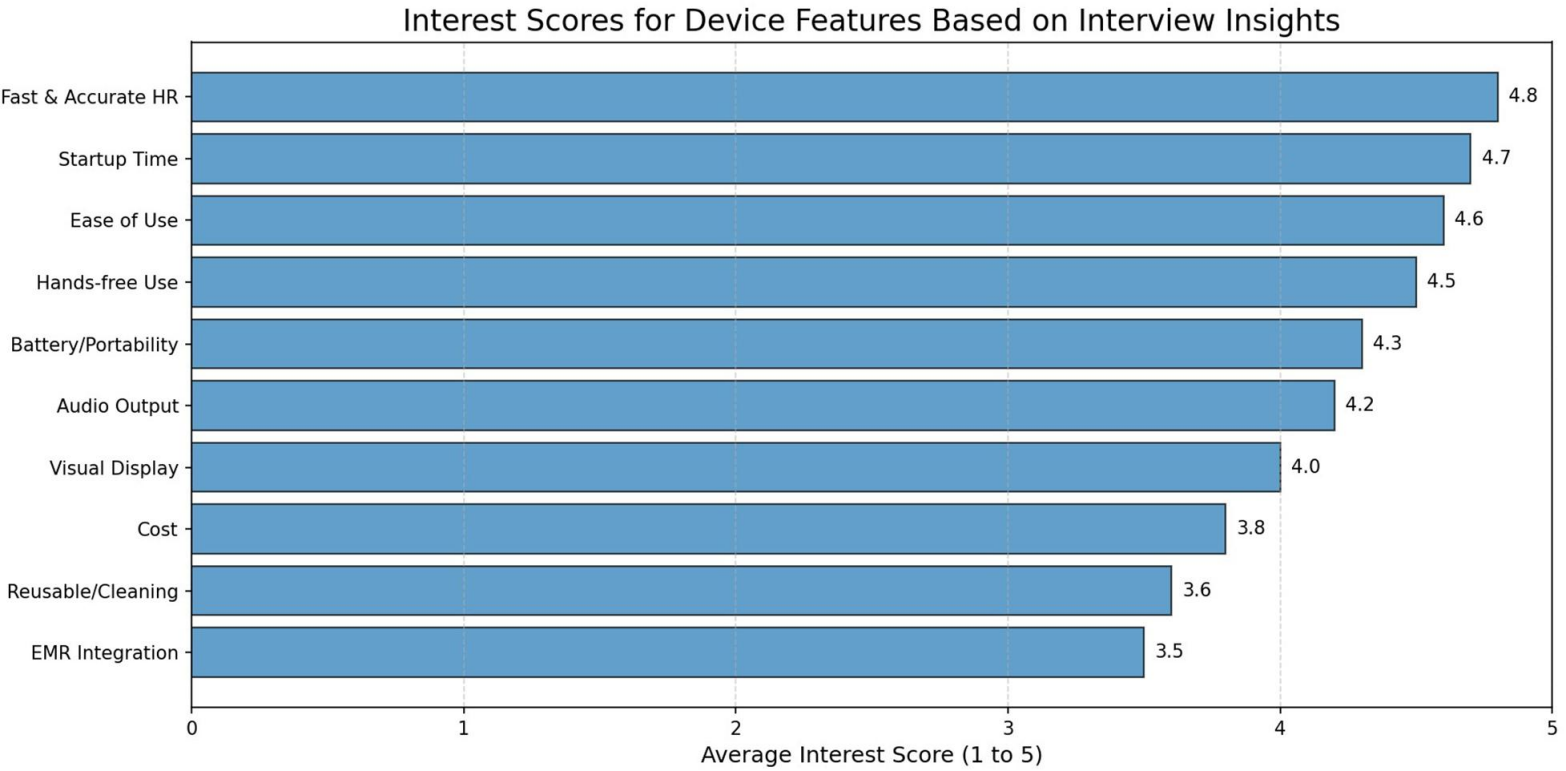
Interest scores for device features.

Regarding HRD functionality, many clinicians reported varying levels of interest that aligned with specific device requirements. As shown in Figure 3, the most important interest score for a new HRD device for neonatal HR assessment was the time of HR display and its accuracy compared with clinical HR monitoring modalities such as ECG and PO.

The second most important interest to examine during iterative development was start-up time, defined as the time needed to turn on and operate the HRD as intended. In line with the golden minute rule, most participants preferred a device capable of providing HR reading within the first few seconds of contact, up to 5–10 s.

The third most important aspect was ease of use. Participants showed a preference for a standalone device (76%) compared with a stethoscope attachment (7%), whereas the rest (17%) were neutral to both approaches. Ease of use also included industrial design features, where the device’s form factor influenced its usability within clinical settings.

Furthermore, participants reported a mean interest score of 4.3 out of 5 for battery life, indicating a preference for a device with a long battery life of up to 1 year to minimize charging requirements. In contrast, the lowest mean interest scores in user requirements were observed for reusability (3.6 out of 5) and EMR integration (3.5 out of 5).

### 3.2 GPT-4o performance metrics

For the overall summarization of all interviews, GPT-4o was able to recognize 27 interviews recorded out of the total 29 interviews conducted (93%). By prompting the GenAI about its mistake, the retrieval was repeated, and all 29 interviews were recognized.

**Figure 4:**
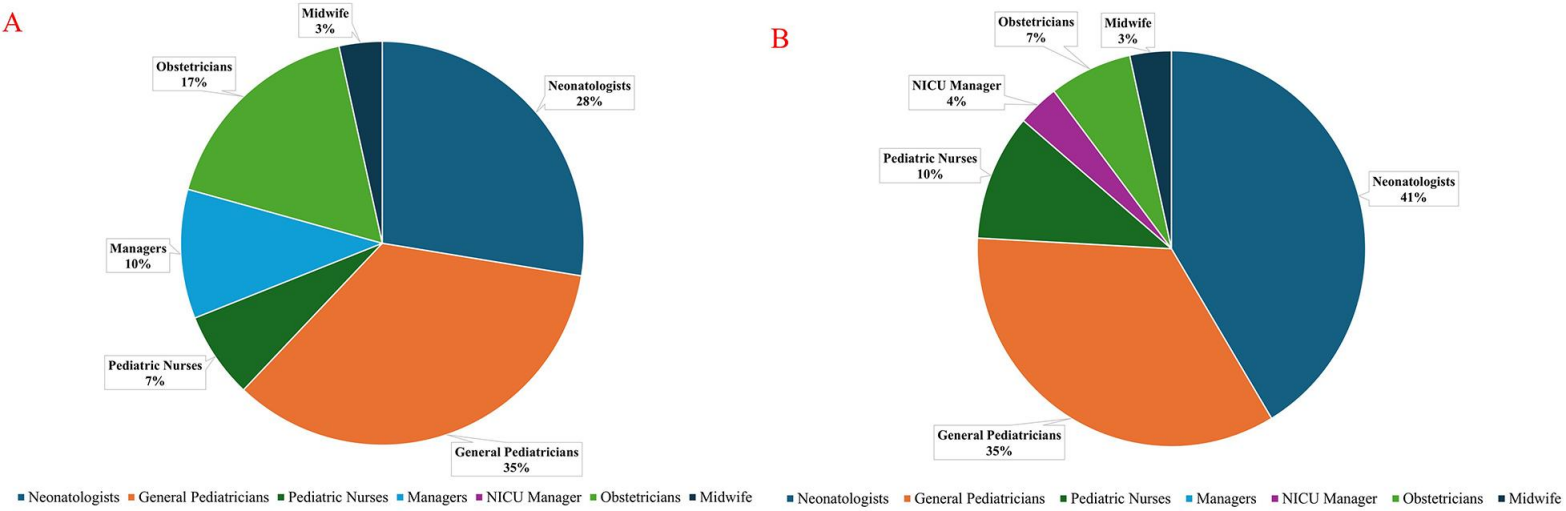
**(A)** GenAI-inferred primary specialities of interviewees. **(B)** True primary specialities of interviewees.

The GenAI also provided the main specialties as 8 neonatologists, 10 general pediatricians, 2 pediatric nurses, 3 managers, 5 obstetricians, and 1 midwife. These outcomes deviated from the ground truth, which identified primary specialities for the interviewee group as 12 neonatologists, 10 general pediatricians, three pediatric nurses, one NICU manager, two obstetricians, and one midwife, as shown in Figure 4. Overall, 10 out of 29 main specialties were incorrectly classified. Each interviewee had diverse roles and experiences throughout their careers, which the GenAI was not able to recognize effectively. In turn, GPT-4o recognized previous experiences that each participant had throughout their career.

Finally, GPT-4o accurately recognized the total years of experience for each interviewee, as well as their primary care setting. The GenAI was able to identify objective values measured in years for each participant accurately, achieving 100% similarity to ground truth annotations.

## 4 Discussion

This study provides a framework for conducting user-requirement interviews and extracting key insights from these interviews using GenAI tools. It explores how these requirements can be curated to support the iterative development of a pediatric medical device capable of providing quick and accurate HR measurements for neonatal resuscitation. It was evident that all 29 medical professionals interviewed confirmed the clinical gap in the current HR measurement modalities for neonatal care and recognized this issue as important.

The faster I know if the baby has a pulse, the quicker I can start compressions or continue ventilation.—Neonatologist 13

### 4.1 Use cases insights

Participants’ perspectives on the use of an HRD for neonatal resuscitation varied depending on their clinical environment, experience, and specialty. For example, obstetricians believed that the HRD could be used during C-sections where newborns may appear pale and often do not cry immediately, leading to physician stress during delayed cord clamping. An inattentive increase in the newborn’s blood volume may increase the risk of jaundice. Therefore, obstetricians believed that rapid HR estimation could validate their delayed cord-clamping decisions while limiting clinical risks. Most participants supported the use of the HRD by non-specialists in rural hospitals and medical centers with limited direct access to pediatric subspecialists. They believed that this technology can deliver a higher standard of care regardless of the physician’s experience in neonatal care, especially in rural settings.

If a midwife or a student can use it without second guessing, then you’re onto something.—Midwife 19

A surprising insight shared by most interviewees was the frustration with ECG electrode placement on newborns. They conveyed that wet electrodes slip due to the softness and fragility of neonatal skin, requiring the use of tape to hold electrodes in place. This insight was considered surprising, as this issue was not framed as a niche problem but as a common frustration among interviewees. Interviewees highly valued the use of dry ECG electrodes rather than the current gold-standard wet electrodes. The identified concern regarding the use of dry electrodes in the clinical setting was their inability to obtain reliable ECG signals suitable for clinical diagnostics.

Dry contact is great in theory, but unless the signal is strong enough to overcome noise, especially in the first minute, it’s not ready.—Neonatologist 18

The use of new sensor types for ECG acquisition requires systemic changes that enable clinicians to learn the new technology before implementing it in their respective practices. This can be facilitated through educational programs that demonstrate the value of new ECG acquisition technologies that have been clinically tested using randomized trials and research studies.

It was evident that this GenAI-based framework for extracting use-case insights from interviews is both feasible and capable of offering valuable information about clinical problems, as well as providing an in-depth understanding of clinician frustrations with current state-of-the art technologies. Also, the diverse use-case scenarios discussed with physicians allowed us to consider the secondary and tertiary applications for a neonatal HRD within the pediatric domain. Secondary use cases include support for delayed cord-clamping decisions to assist in cord-clamping timing, intermittent HR monitoring postdelivery for routine checkups, and triage support in low-resource settings to guide non-specialists in care delivery. Broader tertiary use cases include training and simulation for teaching NRP protocols to residents, support for clinical documentation in patient charting, and quality improvement audits by using time-stamped HR information to review workflow and adherence to NRP guidelines.

### 4.2 HRD development insights

On the other hand, the interviews revealed valuable information about important device features that should be considered to support wide device adoption.

Firstly, the participants requested that the proposed HRD be examined through formal, research-backed validation, including proof-of concept validation studies and the curation of HR datasets to support research dissemination.

If it’s not as accurate as ECG, then people are not going to trust it—especially in the NICU where we’re trained to rely on ECG first.—Neonatologist 3

The data has to be published. If you want uptake, people will ask: how does this compare to ECG? To NRP? You can’t just say it’s good—you need studies.—Neonatologist 18

These aspects would facilitate clinician acceptance of the device because they can rely on research validating its intended use in neonatal resuscitation. Clinical studies and ensuing publications on this device provide preliminary support for meeting these clinical acceptance requirements.

From a device perspective, participants preferred an easy-to-use device that did not require significant training. The device should also feature a rugged fluid-resistant design capable of withstanding drops, frequent sterilization with antiseptic chemicals, and exposure to neonatal fluids.

If it takes more than a couple of seconds to turn on, I’ll probably default to what I’m already using.—Neonatologist 6

“In NICU, infection control is a big deal. If we’re reusing this, it has to be super easy to clean.”—Neonatologist 16

Participants also depressed a preference for having a device available in each delivery room rather than the one that is being carried around. This preference was justified due to concerns regarding misplacement or theft. Device’s battery life was another key point of interest for many participants. Interviewees requested a device that did not need to be recharged and could be used for up to 1 year. These requirements arose from current issues that these clinicians encounter with battery-powered devices, which are usually forgotten to be charged and/or become inoperable during use due to battery issues.

Another surprising insight was the participant’s preference for EMR and seamless clinical workflow integration. Participants stated that HRD integration with EMR systems can enable objective HR recordings. These recordings could inform future medical decision-making for the newborn. Also, the recordings could be used to further mitigate medicolegal and insurance issues related to neonatal resuscitation.

If a baby doesn’t make it and someone says “why didn’t you use ECG?” —you need to be sure this device is defensible. That’s the medicolegal reality.—Neontologist 13

All the abovementioned insights showed that interviewees prefer a standalone HRD for neonatal resuscitation that offers long battery life (up to 1 year), features a waterproof design, is easy to use, and is capable of providing clinical HR measurements using dry electrodes within a few seconds of contact. It is important to note that this study serves as a precursor to the iterative development of an infant HRD that has been validated clinically for neonatal resuscitation and incorporates many of the identified user requirements. However, certain features, such as EMR integration and prolonged battery life of up to 1 year, were not implemented during development because these features required significant technical resources that were not available during development. It is evident that the features of the current prototype device align closely with clinician-driven insights, demonstrating that the proposed framework is successful in translating clinician needs into coherent device requirements.

### 4.3 GPT-4o discrepancies

Furthermore, this study investigated the information retrieval mechanisms of GPT-4o that enable it to generate complex responses based on chat history. In this case, we determined the capabilities of GenAI for summarizing the abovementioned interviews and producing quantitative outcomes from the entire chat. These results were compared with manual annotations, which act as the ground truth in the study. For example, the GenAI initially failed to recognize all the interviews that were conducted. GenAI was able to recognize 27 of the 29 total interviews. This example suggests that information retrieval from long chat histories may be incomplete in extended dialogs. As the chat dialog grows, earlier content may fall outside the effective context window of the model during inference. This process can lead to omissions and degradation in counting and coverage-based tasks.

This showed that information retrieval may not be optimal for small- to large-scale recollection, which the user should consider when using GenAI for total interview statistical analysis. After the recognition of the interviews, the GenAI was prompted to examine and categorized the specialties of the interviewees.

This difficulty in recognizing primary specialties using GenAI can be attributed to the complexity of participants’ career trajectories over time. Medical professionals with extensive experience often hold multiple roles across clinical care, leadership, education, and research, making it challenging for GenAI to infer a single primary specialty from interview text alone. This limitation suggests that GenAI-derived specialty classifications from semistructured interviews should not be used independently for quantitative, category-dependent metrics without verification. Specialty information should be captured through structured self-reporting and/or validated through a human-in-the-loop review process before analysis.

Overall, GenAI could be used to summarize and extract valuable insights from long, semistructured interviews. However, it is important for information curators to understand the limitations and constraints of current LLMs. These constraints are evident in their retrieval mechanisms, where specific prompt engineering is required to ensure accurate extraction of all the required information. Prompt engineers and user-requirement researchers must prepare highly specialized prompt templates, aligned with their template interview questionnaires, to get the most benefit from LLM-based summarization. It is obvious that key user requirements for a pediatric medical device can be extracted from anonymized transcripts. Furthermore, LLMs can be used to extract key findings from clinician interviews to inform early-stage HRD design and iterative development. This work acts as a foundation for understanding the application of LLMs in user-requirement extraction.

### 4.4 Limitations and future works

This work introduced a framework for conducting semistructured interviews to develop a pediatric medical device. The methodology used GenAI to validate the clinical problem, summarize interview transcripts, and extract important key user-requirement insights. With these in mind, the study is limited by its focus on a single LLM model, GPT-4o, which may introduce LLM bias. Further work should explore the use of medically specialized LLMs to observe the differences between general and specialized LLMs in user-requirement extraction. In addition, future studies should explore a broader range of LLMs and model versions, such as DeepSeek V3 (DeepSeek), Claude (Anthropic), Gemini (Google), Llama (Meta AI), and newer models of OpenAI’s ChatGPT series such as GPT-5 mini and GPT-5.2.

Prompt chaining was used to extract information from each interview. This approach may introduce dependency effects, such as context carryover, which can influence later responses and affect interpretability. Future work should compare chain prompting approaches with isolated, single-shot prompting approaches, as well as the use of separate chats for each task, to quantify differences in extracted insights and user requirements.

It is important to note that the use of a single principal reviewerfor user extraction in this study may introduce potential bias. The findings may reflect reviewer subjectivity and model-dependent interpretation, especially for open-ended highlights, captured by prompt item 7. However, this framework was presented for early-stage, user-centered HRD design, where the primary goal is to demonstrate how clinician interviews and LLMs can be used to capture and synthesize clinician-driven device features. The study did not explore inter-rater reliability. Future work should explore strategies to mitigate these potential biases, including independent coding, follow-up interviews, and clinician focus groups, to further refine HRD design, ultimately leading to a higher likelihood of clinical acceptance.

Another limitation of this work was its focus on pediatric device development. This narrow scope may limit the use of the framework in the pediatric settings. However, this study could serve as a foundation for other researchers and device developers to explore modifications to this framework for medical device development in other areas, such as surgical procedures and patient remote monitoring.

Finally, future researchers in this field must address the security and privacy risks associated with GenAI to collect and analyze user perspectives for qualitative research that informs medical device design. From a clinician–interviewee perspective, key concerns include the inadvertent disclosure of identifiable and/or sensitive information contained in interview transcripts, such as names and institutions. Also, the privacy risks involve the transparency over how their statements are retained, reused, or shared through GenAI. To mitigate these risks, future researchers should apply deidentification and data anonymization before LLM use and restrict data access through role-based permissions. Also, researchers should use secure, and approved hosting servers with encryption. Researchers should also strengthen governance by ensuring explicit consent language and placing safeguards that prevent the regeneration of personal identifiers to maintain the value of GenAI-assisted synthesis for early-stage device design and development.

## 5 Conclusion

The purpose of this study was to provide a GenAI framework for acquiring user requirements to support the iterative development of pediatric medical devices. For this study, we focused on the development of a neonatal HR monitor for use in neonatal resuscitation to demonstrate the feasibility of the proposed framework and to derive key insights relevant to neonatal care for pediatric researchers. We interviewed 29 medical professionals and used LLMs to find key insights that informed the medical device developmental process. LLMs are tools that can assist researchers in transcribing and coding prerecorded interviews and extracting important information from these interviews. In this study, we explored the use of GPT-4o. The LLM operated accurately in identifying quantitative, and qualitative data across all interview sessions, recognizing the main themes of user-requirements. It is important to note that the LLM operated on anonymized transcripts derived from semistructured interviews. This process allowed us to investigate the true capabilities of LLMs in moderately complex interview conversations.

Through this study, the recognized user-requirement themes included functionality features such as fast and accurate HR measurement, display, battery life, EMR integration, and start-up time. In addition, industrial design features informed themes related to the ease of use, portability, and reusability. These user requirements were later used in the early-stage development of an infant HRD, which was clinically validated in accordance with the recommendations from participants. Device development utilized these curated user requirements to inform design concepts and ensure suitability for mass clinical adoption. This study offers an interview framework and demonstrates the application of GenAI tools for seamless user-requirement curation and user insight generation in neonatal medical device development. It is evident that LLMs can serve as reliable tools for interview transcription and key insight extraction to support the development of medical devices.

## Data Availability

The raw data supporting the conclusions of this article will be made available by the authors, without undue reservation. The conversational chat used to extract insights from the interviews for this study.
