# Supplementary material for: A framework for generative AI-driven extraction of clinical user needs in pediatric device development
Source: Front Digit Health. 2026 Apr 14;8:1726098. doi: 10.3389/fdgth.2026.1726098 (PMC13121137; doi:10.3389/fdgth.2026.1726098)
Supplement: Supplementary file 1 [file Datasheet1.pdf]

S1 Appendix: COREQ Checklist

**No. Item**	**Guide questions and descriptions**	**Reported on Page #**
**Domain 1: Research team and reflexivity**		
Personal Characteristics		
1. Interviewer/facilitator	Which author/s conducted the interview or focus group?	Page 5/Lines 150-156
2. Credentials	What were the researcher's credentials? E.g., PhD, MD	Page 5/Lines 150-156
3. Occupation	What was their occupation at the time of the study?	Page 5/Lines 152-158
4. Gender	Was the researcher male or female?	Male
5. Experience and training	What experience or training did the researcher have?	Page 5/Lines 152-158
Relationship with participants		
6. Relationship established	Was a relationship established prior to study commencement?	Not reported **No**
7. Participant knowledge of the interviewer	What did the participants know about the researcher? e.g. personal goals, reasons for doing the research	Interviewees are informed on the purpose of the interview and gave informed consent
8. Interviewer characteristics	What characteristics were reported about the inter viewer/facilitator? e.g. Bias, assumptions, reasons and interests in the research topic	Facilitator/interviewer encountered the stated problem and is validating the need. Page 5/Lines 154-157

**Domain 2: study design**		
Theoretical framework		
9. Methodological orientation and Theory	What methodological orientation was stated to underpin the study? e.g. grounded theory, discourse analysis, ethnography, phenomenology, content analysis	Content Analysis
Participant selection		
10. Sampling	How were participants selected? e.g. purposive, convenience, consecutive, snowball	Page 5\Lines 161-162
11. Method of approach	How were participants approached? e.g. face-to-face, telephone, mail, email	Email
12. Sample size	How many participants were in the study?	29 participants<br>Page 4\Lines 137-140
13. Non-participation	How many people refused to participate or dropped out? Reasons?	None
Setting		
14. Setting of data collection	Where was the data collected? e.g. home, clinic, workplace	Pages 4-5/Lines 148-150
15. Presence of non-participants	Was anyone else present besides the participants and researchers?	**No**
16. Description of sample	What are the important characteristics of the sample? e.g. demographic data, date	Page 4/ Lines 137-138
Data collection		
17. Interview guide	Were questions, prompts, guides provided by the authors? Was it pilot tested?	Page 4/Lines 141 - 146
18. Repeat interviews	Were repeat inter views carried out? If yes, how many?	**No**
19. Audio/visual recording	Did the research use audio or visual recording to collect the data?	Pages 4-5/Lines 148-150
20. Field notes	Were field notes made during and/or after the interview or focus group?	During
21. Duration	What was the duration of the interviews or focus group?	Page 4/Line 146
22. Data saturation	Was data saturation discussed?	N/A
23. Transcripts returned	Were transcripts returned to participants for comment and/or correction?	No. The transcripts are provided to the researcher/interviewer for validation and correction
**Domain 3: analysis and findings**		
Data analysis		

24. Number of data coders	How many data coders coded the data?	**One** Page 5/Lines 158-159
25. Description of the coding tree	Did authors provide a description of the coding tree?	**Coding Tree provided as template prompts, outlining the acquired theme information.**
26. Derivation of themes	Were themes identified in advance or derived from the data?	**Some themes were derived from the data and other themes were identified in advance**
27. Software	What software, if applicable, was used to manage the data?	**N/A**
28. Participant checking	Did participants provide feedback on the findings?	**No**
Reporting		
29. Quotations presented	Were participant quotations presented to illustrate the themes/findings? Was each quotation identified? e.g. participant number	Pages 8-10
30. Data and findings consistent	Was there consistency between the data presented and the findings?	**Yes**
31. Clarity of major themes	Were major themes clearly presented in the findings?	Pages 6-7. **Yes, themes are identified as device features.**
32. Clarity of minor themes	Is there a description of diverse cases or discussion of minor themes?	Pages 8-10 **Yes, minor themes are considered surprising qualitative insights into use cases and device features.**
