# Supplementary material for: A framework for generative AI-driven extraction of clinical user needs in pediatric device development
Source: Front Digit Health. 2026 Apr 14;8:1726098. doi: 10.3389/fdgth.2026.1726098 (PMC13121137; doi:10.3389/fdgth.2026.1726098)
Supplement: Supplementary file 2 [file Datasheet2.pdf]

S2 Appendix: User-requirement Questionnaire

User-Requirement Questionnaire:

• How long have you been practicing as a medical professional?

• What do you specialize in?

• What setting do you work under?

• How many hospitals or clinics are you attached to?

• How many patients do you see in your primary place of practice?

• How Many patients do you seen per week (combining all places of practice)?

• What is the patient capacity in your primary place of practice?

• What is the combined patient capacity in all places of practice?

• What products do you use in your routine work process (analog or digital stethoscopes)?

• What products do you currently use in diagnostics during delivery?

• Is this facility standardized on one brand of products? Yes or No

• Does this facility provide different diagnostic products for infant diagnostics? __ Yes __ No

• What are the primary factors that affect your choice of products?

Most important: ______________

2nd important: ______________

3rd important: ______________

• Who is normally involved in the selection of diagnostic products used by this facility? Is there an evaluation process? Who is involved in the evaluation?

• Are you familiar with or have you heard about any solutions for diagnostics

for infants? Yes __ No __

• What would you say are the primary reasons why a device like the HRD is not currently used in this facility?

Discussion of infant HRD Concept for Neonatal Resuscitation Efforts:

High-Level Device Overview:

- Measures and displays Heart Rate quickly and accurately when placed on infant's chest
- Uses ECG technology
- Use Case
    - Delivery of infant
    - Neonatal Resuscitation

Without the benefit of additional conversation/questions, assuming that this HRD device works as advertised AND the incremental expense is justified, what would be your interest in integrating it in your routine/process at this facility?

1. Definitely not

2. Probably not

3. Unsure at this time

4. Probably

5. Definitely

• What are the top 1 or 2 reasons for assigning this rating?

• What do you think would be the main concerns, if any, about implementing this type of device in your workflow or at this facility?

• Is there anything that you can think of that you would suggest to change the way this type of device might be used to make it more appealing?

• If you were to consider adopting an HRD, how do you think you would implement this type of system:

(Don't prompt unless necessary)

Pilot would be required __

Start with one facility or multiple facilities __

Start with a few doctors at one facility and gain experience? __

Use by a few doctors at other facilities? __

Expand to other healthcare personnel use? __

Eventually expand use to all healthcare personnel at all facilities? __

Other __

• Let's talk about the suggested benefits of this device. How important do you think each of these would be in your facility (Rate: 1= not interested/ not important at all, 2= slightly interested/not too important, 3=moderately interested/somewhat important, 4= very interested/important, 5 = definitely interested/very important)

Feature Rating

Potential increase in the speed of diagnostics for infants __

Enable doctors to have more time for undertaking life saving measures __

Potential to reduce incidence of skin irritation requiring medical attention __

Consistency in diagnosing rhythm for all infants __

Reduction of errors in diagnosing infants __

Ability to automatically document all readings during post-partum care __

Reduction in potential for liability resulting from data capture __

Potential to position facility as "best in class" care using latest available technology __

• Are there any other potential benefits (or concerns) that have not been dis-

cussed? Are there any questions about this device that have not been answered?

• In addition to the benefits previously identified, if the iHRD additionally offered integrations with patient medical records, how would you rate your interest in integrating this product in your facility?

Definitely not __

Probably not __

Unsure at this time __

Probably __

Definitely __

END
